# LIFITEGRAST 5% FOR DRY EYE DISEASE: COMBINED EFFICACY AND SAFETY FROM FIVE RANDOMIZED CONTROLLED TRIALS

**DOI:** 10.1093/geroni/igz038.983

**Published:** 2019-11-08

**Authors:** Agustin L Gonzalez, Eric Donnenfeld, Christophe Baudouin, Edward Holland, Kelly Nichols, Paul Karpecki, Mohamed Hamdani, Amir Shojaei

**Affiliations:** 1 Eye and Vision North Texas Center for Dry Eye and Ocular Surface Disease, Richardson, Texas, United States; 2 Ophthalmic Consultants of Long Island, Garden City, New York, United States; 3 Quinze-Vingts National Ophthalmology Hospital, Paris, France; 4 Cincinnati Eye Institute-Northern, Edgewood, Kentucky, United States; 5 University of Alabama, Birmingham, School of Optometry, Birmingham, Alabama, United States; 6 Kentucky Eye Institute, Lexington, Kentucky, United States; 7 Apellis Pharmaceuticals, Crestwood, Kentucky, United States; 8 Takeda, Vice President, Global Head - Ophthalmology, Lexington, Massachusetts, United States

## Abstract

Five randomized, double-masked, placebo-controlled trials were conducted in the US in adults with DED, an eye dryness score (EDS, visual analogue scale [VAS], 0–100) ≥40, and inferior corneal staining score ([ICSS], 0-4) ≥2.0 at study entry: four 84-day efficacy trials (phase 2, lifitegrast n=58, placebo, n=58; phase 3: OPUS-1, n=293, n=295; OPUS-2, n=358, n=360; OPUS-3, n=355, n=356) and a 1-year safety study (SONATA, lifitegrast n=220, placebo n=111). The pooled population had a mean age of 59.4 years, and 76% were females. Lifitegrast treatment, versus placebo, significantly improved EDS from baseline to day 84 in three of the four trials: OPUS-1 (treatment effect [TE] 4.7; P=0.0311), OPUS-2 (TE 12.3; P<0.0001), and OPUS-3 (TE 7.5; P=0.0003). Lifitegrast significantly improved ICSS in the phase 2 (TE 0.25; P=0.0498) and OPUS-1 (TE 0.23; P=0.0007) trials, and nominally in OPUS-3 (TE 0.17; nominal P=0.0135). The responder analyses from OPUS-2 and OPUS-3 assessed the proportion of subjects with an EDS reduction from baseline (≥10, ≥15, ≥20 points), and percentage change from baseline (≥30, ≥50, ≥70%), to days 14, 42 and 84. More subjects achieved ≥30% EDS reduction with lifitegrast versus placebo in Opus-2, and Opus 3 at days 14, 42, and 84 (all nominal P<0.0001). A similar trend was seen at other response thresholds. Pooled safety data (lifitegrast n=1287; placebo, n=1177) indicated Lifitegrast was well tolerated with no serious ocular adverse events. Lifitegrast significantly improved signs and symptoms of DED in adult subjects, with EDS improvements observed starting at day 14 after treatment.

